# Supplementary material for: Transcriptional analysis of wheat seedlings inoculated with Fusarium culmorum under continual exposure to disease defence inductors
Source: PLoS One. 2020 Feb 11;15(2):e0224413. doi: 10.1371/journal.pone.0224413 (PMC7012390; doi:10.1371/journal.pone.0224413)
Supplement: S1 File — (DOCX) [file pone.0224413.s001.docx]

**S1 Supporting information**

**Confirmation of *F. culmorum* presence in inoculated seeds**

The presence of *F. culmorum* in seeds was confirmed in two ways, using quantitative (real-time) polymerase chain reaction (qPCR) and enzyme-linked immunosorbent assay (ELISA). DNA from 50 mg of inoculated and healthy seeds was isolated using a DNeasy Plant Mini Kit (Qiagen, Hilden, Germany) according to the manufacturer’s instructions. The DNA concentration was measured by Qubit (Thermo Fisher Scientific, Waltham, MA, USA), and the DNA was diluted to 10 ng μl^−1^. Primers and conditions for the qPCR reaction were as described by Moradi et al. [1]. The qPCR solution consisted of 1× SYBR Master Mix (Top-Bio, Prague, Czech Republic), 10 ng DNA, and water to volume 15 μl. The qPCR analyses were performed using the CFX96TM Real-Time PCR Detection System (Bio-Rad, Hercules, CA, USA). The gDNA isolated from mycelium of *F. culmorum* tribe KM16902 with known concentration was used for inspecting the amount of *F. culmorum* DNA in inoculated seeds. The second way of confirmation was to measure mycotoxin content by ELISA in both inoculated and non-inoculated seeds. For the ELISA method, R-Biopharm AG kits (Darmstadt, Germany) were used. A combination of RIDASCREEN^®^DON and RIDACREEN^®^FAST DON kits was used to determine DON. The limit of quantification for DON was 20 μg kg^−1^.

1. Moradi M, Oerke EC, Steiner U, Tesfaye D, Schellander K, Dehne HW. Microbiological and SYBR green real-time PCR detection of major Fusarium head blight pathogens on wheat ears. Microbiology. 2010; 79(5):646–654. doi:10.1134/S0026261710050097
